# Development and validation of World Health Organization Assessment Schedule 2.0 for persons with disabilities having community rehabilitation services in Hong Kong

**DOI:** 10.1371/journal.pone.0326409

**Published:** 2026-08-06

**Authors:** Tiffany Ching Man Choi, Yin Kin Lee, Ho Lim Lee, Pandora Chao Ming Wang, Arran Siu Lun Leung, Herman Mun Cheung Lau, Eric Lu Shek Chan

**Affiliations:** 1 S.K. Yee School of Health Sciences, Saint Francis University, Hong Kong Special Administrative Region of the People's Republic of China, China; 2 Services for People with Disabilities, Christian Family Service Centre, Hong Kong Special Administrative Region of the People's Republic of China, China; University of Perugia: Universita degli Studi di Perugia, ITALY

## Abstract

**Background:**

The World Health Organization Disability Assessment Schedule 2.0 (WHODAS 2.0) is a generic instrument aligned with the International Classification of Functioning, Disability and Health (ICF). Existing Chinese versions are not fully applicable to the Hong Kong sociolinguistic context. This study aimed to translate, culturally adapt, and preliminarily evaluate the psychometric properties of a Hong Kong Traditional Chinese version of WHODAS 2.0 (WHODAS 2.0 TC-HK).

**Methods:**

WHODAS 2.0 was translated using forward–backward procedures. A convenience sample of 177 adults with disabilities receiving community rehabilitation services completed WHODAS 2.0 TC-HK, the SF-36 TC-HK, and WHOQOL-BREF TC-HK. Internal consistency, test–retest reliability, convergent and concurrent validity were examined. Construct validity was explored using confirmatory factor analysis (CFA) based on the original six-domain model.

**Results:**

Internal consistency was good across domains (Cronbach’s α = 0.86–0.89). Test–retest reliability was moderate to good (ICC = 0.59–0.87). WHODAS 2.0 TC‑HK showed moderate negative correlations with SF‑36 TC‑HK and WHOQOL‑BREF TC‑HK domains, supporting convergent and concurrent validity. Confirmatory factor analysis of the 32‑item version demonstrated suboptimal model fit, indicating only partial support for the original factor structure.

**Conclusions:**

WHODAS 2.0 TC‑HK demonstrates good reliability and acceptable convergent and concurrent validity among adults receiving community rehabilitation services in Hong Kong. However, structural validity of the instrument remains preliminary and requires further investigation in larger and more diverse samples, particularly for the complete 36‑item version.

## Introduction

According to the International Classification of Functioning, Disability and Health (ICF), functioning and disability are a result of the interaction of an individual’s health condition and contextual factors (environmental and personal factors) [1]. The ICF classification of disability has shifted from the previous medically focused definition to a biopsychosocial approach [2]. This complex, dynamic, multidimensional, and contested nature of the concept of disability increases the challenges of disability measurement [3]. Whilst the ICF framework appreciates the interaction between different factors that affect disability and functioning, its complexity in linking the standard instrument of disability measurement to the ICF model conceptually and operationally makes direct implementation in clinical practice challenging [4]. A variety of ICF-based measurement tools were therefore derived, attempting to develop a standardised procedure to collect disability and functioning data. The World Health Organization Disability Assessment Schedule 2.0 (WHODAS 2.0) was invented in 2010 [5,6]. It is a 36-item self-administered tool designed to assess functioning in six life domains. The WHODAS 2.0 acted as a generalised and cross-cultural method to measure individuals’ limitations on different activities and social participation in a way that is independent of medical diagnosis [7]. It has been used in cross-country research for general populations and those with physical, mental, and neurological conditions [5,8,9].

The 36-item WHODAS 2.0 has been validated with adequate reliability in various groups, including arthritis, stroke, chronic psychotic disorder, depression, and back pain [10–13]. The WHODAS 2.0 supersedes WHODAS II and shows many advantages for example used across all diseases including mental, neurological and addictive disorders; short, simple and easy to administer; applicable in both clinical and general population settings; producing standardized disability levels and profiles; applicable across cultures, in all adult populations and directly linked at the level of the concepts to ICF [6].

WHODAS 2.0 has been translated into around 50 languages and dialects and is used in almost 30 areas of research in around 100 countries [6]. Experts in Taiwan once modified and translated WHODAS 2.0 to an instrument called Functioning Disability Evaluation Scale (FUNDES), which was in Traditional Chinese with an excellent internal consistency (Cronbach’s alpha: 0.9); however, it was not applicable in Hong Kong communities as that version included two new domains which were not shown in the English original version [14].

A simplified Chinese version of WHODAS 2.0 has also been developed. However, this version may not be completely applicable in Hong Kong since Cantonese (Hong Kong Chinese, in the form of Traditional Chinese) is the most common form/dialect of spoken Chinese used by the Hong Kong population, and there were cultural differences in language semantics, idiomatic expressions and concepts [15].

In Hong Kong, the government highly recommended the application of ICF in clinical assessment, collecting statistics, formulating rehabilitation plans, and prioritising social services as mentioned in the Persons with Disabilities and Rehabilitation Programme Plan in 2020 [16]. Yet, there is no generic assessment tool revealing the disability status for local persons with disabilities in community services. Many assessment instruments mainly focus on functional activities such as walking, eating, dressing, and grooming instead of social participation and activities [8]. Also, service users cannot participate and express their concerns in the rehabilitation plan [17]. The adaptation of the WHO standardized measurement tool with the ICF framework in Hong Kong, WHODAS2.0 TC-HK, seems to be a more feasible way to understand the disabilities and the needs of service users in community services with the multi-disciplinary care model.

Therefore, the purpose of this study was to adapt and validate a Hong Kong Chinese version of WHODAS 2.0 for persons with disabilities receiving community services in Hong Kong.

## Methods

### Subject recruitment

A convenient sampling technique was used. 177 clients with disabilities were recruited from four units subsidized by the Social Welfare Department SWD, Hong Kong Government, and held by a local non-governmental organization (NGO). The total service boundaries covered by the four units were around 48% of persons with disabilities in Hong Kong [18].

The following types of disabilities were included in the study: (1) physical disability; (2) hearing impairment; (3) visual impairment; (4) speech impairment; (5) mental illness; (6) intellectual disability (ID); (7) autism, attention deficit hyperactivity disorder (ADHD) and specific learning difficulties; and (8) chronic illness/visceral disability.

Inclusion criteria were: (1) be able to have basic communication and understandings the questions; (2) having no major hearing difficulties; (3) be willing to participate in the study; (4) be active users having at least one service in one month at the corresponding unit [19]. Exclusion criteria were: (1) with a diagnosis of moderate and severe ID; (2) severe hearing impairment. The purpose of the study was explained to them. An information sheet of the study was distributed, and they were required to sign a consent form. A copy of the consent form was given to each participant.

For the recruitment procedures, potential participants were recruited from four rehabilitation units from the “Christian Family Service Centre (CFSC)”, which serves the community with the motto “Improving quality of life, uniting clients and community”.

The first unit was “Community Rehabilitation Day Centre (KRD)”, which mainly serves clients with physical disabilities and chronic illnesses living in Kowloon, Hong Kong. It provided exercise training on a day centre basis. Active users were 225, and 14 subjects were recruited in KRD.

The second unit was “Cheerful Place - District Support Centre - Kwun Tong East (DSC)” which provided day care service and day respite care on a centre basis for clients with ADHD, ID, speech impairment, and visual impairment living in Kowloon East, Hong Kong. Active users were 450, and 33 subjects were recruited in DSC.

The third unit was “Everjoy (RHCS)” which offers home care services for clients with severe disabilities on an outreach basis in their home. Rehabilitation services, personal care services, and nursing care were provided for those with physical disability, ID, ADHD, and chronic illness/visceral disability living in Kowloon East, Hong Kong. Active users were 550, and 88 subjects were recruited in RHCS.

The last unit was an outreach team for private residential care homes (POT) serving clients with mental illness, physical disability, ID, ADHD, hearing impairment, and visual impairment. Rehabilitation services and recreational activities were provided for these clients in private hostels in Kowloon and Hong Kong Island, Hong Kong. Active users were 280, and 42 subjects were recruited in POT.

The study period was from January 2023 to December 2024. The official permission to use WHODAS 2.0 was granted by WHO in April 2025. The study was approved by the Research and Ethics Committee of the Saint Francis University, Hong Kong (HRE220182).

### Translation process

The initial phase of the study included translation and adaptation of the WHODAS 2.0 from its original version in Hong Kong Chinese, the commonest form/dialect of spoken Chinese used by the Hong Kong population.

The standard “forward-backward” procedure was adopted to translate the English WHODAS 2.0 to Hong Kong Chinese to ensure both the accuracy of meaning and cultural acceptability. The forward translation was performed in close collaboration with experienced healthcare professionals in the care of clients with disabilities. This version was blindly back-translated to English by a bilingual native English speaker with a healthcare background.

The two versions of the WHODAS 2.0 were then assessed and compared by a group of healthcare professionals experienced in the care of clients with disabilities and independent linguistic experts. These experts modified the Hong Kong Chinese version when there were differences in meanings between the two versions until a final working Hong Kong Chinese version was approved.

A pilot test of the WHODAS 2.0 TC-HK was completed on 10 clients with disabilities to make sure they could understand and complete all the items.

### Data collection

The second phase of the study involved data collection to examine the validity and reliability of the WHODAS 2.0 TC-HK in persons with disabilities undergoing Community Rehabilitation Services.

### Demographics and disability characteristics

Participants’ characteristics, including age, gender, marital status, education level, and employment status, while disability characteristics, including types of diagnosed disabilities and the presence of co-morbidity (more than one type of disability), were collected for further data analysis.

During the rehabilitation training, participants were asked to complete the WHODAS 2.0 TC-HK, Hong Kong Chinese version of 36-Item Short Form Health Survey (SF-36 TC-HK), and Hong Kong Chinese version of the World Health Organization Quality of Life Abbreviated (WHOQOL-BREF TC-HK).

### Hong Kong Chinese version of World Health Organization Disability Assessment Schedule (WHODAS 2.0 TC-HK)

WHODAS 2.0 TC-HK, a 36-item self-administered version, is an instrument to assess functioning in six life domains: Understanding and communicating (6 items), Getting around (5 items), Self-care (4 items), Getting along with people (5 items), Life activities and Participation in society (8 items). Participants were required to score their level of difficulty in the past 30 days using a 5-point scale (0 = no difficulty to 4 = extreme difficulty or cannot do). The higher the score, the greater disability was.

### Hong Kong Chinese version of 36-Item Short Form Health Survey (SF-36 TC-HK)

SF-36 TC-HK is to measure generic health status and has been widely used in the past two decades in local clinical settings [20–21]. A 36-item self-report measure of health-related quality of life (HRQOL) was also collected. Eight subscales are measuring different domains of HRQOL: physical functioning (PF), role-physical (RP), bodily pain (BP), general health (GH), vitality (VT), social functioning (SF), role-emotional (RE), and mental health (MH). The higher the scores, the better the health and functioning.

### Hong Kong Chinese version of the World Health Organization Quality of Life Abbreviated (WHOQOL-BREF TC-HK)

The WHOQOL-BREF, an abbreviated version of the World Health Organization Quality of Life (WHOQOL)-100 instrument, was also collected. It was developed by WHO to be the generic quality of life assessment [22]. WHOQOL-BREF TC-HK was commonly used to measure quality of life in local studies and acted as the gold standard [23,24]. It consists of 26 items covering four quality-of-life domains: physical (seven items), psychological (six items), social (three items), and environmental (eight items), with two more general questions about health and quality of life. It has a Likert response scale varying from 1 to 5, and scores for each domain, as well as a total score, can be calculated. These scores were represented along a linear scale from 0 to 100, where higher scores reflect better quality of life.

### Statistical analysis

Descriptive statistics were used to summarize demographic data. Each score in outcome measures was calculated based on the formula.

The IBM SPSS Statistics for Windows, version 27.0 (IBM Corporation, Armonk, NY) was utilized for data analysis. The level of statistical significance was set at p ≤ 0.05.

To assess convergent validity, Spearman’s correlation coefficient (rs) was used to investigate the associations between WHODAS 2.0 TC-HK and SF-36 TC-HK. To assess concurrent validity, Spearman’s correlation coefficient (rs) was used to investigate the associations between WHODAS 2.0 TC-HK and WHOQOL-BREF TC-HK. The interpretation rs value was lower than 0.25 as small, 0.25–0.50 as moderate, 0.50–0.75 as good; higher than 0.75 as excellent [25]. To assess reliability, the internal consistency of the WHODAS 2.0 TC-HK was estimated by Cronbach’s alpha coefficient; an alpha score of 0.8 or above was considered good [26]. Test-retest reliability was assessed by Intraclass correlation coefficient (ICC),0.5–0.75 as moderate reliability, 0.75–0.90 as good reliability, and higher than 0.9 indicating excellent reliability [27]. Furthermore, the construct validity of the WHODAS 2.0 TC-HK was tested by confirmatory factor analysis (CFA) to check whether the original hypothesised structure of the WHODAS 2.0 was fitted. To define a satisfactory fit of the model, the cut-off of root means square error of approximation (RMSEA), Goodness of Fit Index (GFI), and Standardized Root Mean Square Residual (SRMR) should be lower than 0.08, higher than 0.90, and lower than 0.08 respectively [28–30]. For the item factor loadings in CFA, it was analysed as ≥0.71 (excellent), 0.63–0.70 (very good), 0.55–0.62 (good), 0.45–0.54 (fair), 0.32–0.44 (poor), and<0.32 (unacceptable and deleted from factor) [31].

## Results

Data from 177 participants were collected and analysed, with subgroups categorized as KRD (n = 14), DSC (n = 33), RHCS (n = 88), and POT (n = 42). Thirty-six participants completed the WHODAS 2.0 TC-HK twice in two different sessions to evaluate the test-retest reliability. Table 1 showed the detailed distribution of age, gender, education, work status, and types of disabilities. The mean age of participants was 55 years (SD ± 15.1). The RHCS subgroup had the oldest participants (60.9 ± 14.5 years), while the DSC group was youngest (43.7 ± 13.6 years). A slightly higher proportion was female in the samples (53.1%). About 32.2% (n = 57) of participants were living independently in the community. All POT participants were hospitalized, contrasting with the DSC group, where 57.6% lived independently. The mean education level was 10.6 years (SD ± 5.2).

**Table 1:** Participants’ demographics and types of disabilities for test-retest.

	Total Sample (n = 36)	KRD (n = 5)	DSC (n = 5)	RHCS (n = 10)	POT (n = 16)
Age, Mean (SD)	53.5±(13.6)	53.6±(17.3)	44.4±(10.7)	57.8±(17.1)	53.6±(10.4)
Female	22(61.1%)	4(80%)	2(40%)	4(40%)	12(75%)
Male	14(38.9%)	1(20%)	3(60%)	6(60%)	4(25%)
**Living condition**					
Independent in community	9(25%)	4(80%)	3(60%)	2(20%)	0
Assisted living	11(30.6%)	1(20%)	2(40%)	8(80%)	0
Hospitalized	16(44.4%)	0	0	0	16(100%)
Education years, Mean(SD)	12.0±(4.5)	10.8±(5.2)	11.2±(5.0)	11.4±(5.2)	13.0±(4.0)
**Work status**					
Paid Work	2(5.6%)	0	2(40%)	0	0
Unemployed other reason	2(5.6%)	0	1(20%)	0	1(6.3%)
Unemployed health reason	13(36.1%)	3(60%)	1(20%)	3(30%)	6(37.5%)
Student	1(2.8%)	0	0	1(10%)	0
Keeping house	2(5.6%)	0	0	1(10%)	1(6.3%)
Retired	10(27.8%)	2(40%)	0	4(40%)	4(25%)
Non-paid work	2(5.6%)	0	0	0	2(12.5%)
Self employed	0	0	0	0	0
Other	4(11.1%)	0	1(20%)	1(10%)	2(12.5%)
**Disability**					
Physical disability	8(22.2%)	4(80%)	1(20%)	2(20%)	1(6.3%)
Hearing impairment	0	0	0	0	0
Visual impairment	2(5.6%)	0	0	2(20%)	0
Speech impairment	3(8.3%)	1(20%)	0	1(10%)	1(6.3%)
Mental illness	16(44.4%)	0	1(20%)	2(20%)	13(81.3%)
Intellectual disability	2(5.6%)	0	2(40%)	0	0
ADHD	1(2.8%)	0	1(20%)	0	0
Chronic illness/visceral disability	4(11.1)	0	0	3(30%)	1(6.3%)

Regarding work status, Unemployment due to health reasons was prevalent in KRD (57.1%) and RHCS (31.8%), whereas 30.3% of the DSC group maintained paid employment.

Physical disabilities were the most common, affecting 27.6% (n = 49) of participants, with the highest prevalence in the KRD subgroup (71.4%). Mental illness was reported by 17.5% (n = 31) of participants, with the POT subgroup showing the highest prevalence (66.7%). Chronic illnesses or visceral disabilities affected 19.2% (n = 34) of participants, primarily in the RHCS subgroup (34.1%).

The test–retest period was 1–16 days, and the mean day was 5.9. 24 clients completed the 32-item version (Do5(2): Life activities: work/study domain cannot be filled) while 12 clients completed the 36-item version. Participants’ demographics and types of disabilities were shown in Table 1.

Summary scores and reliability of the WHODAS 2.0 TC-HK, SF-36 TC-HK, and WHOQOL-BREF TC-HK were shown in Table 2. WHODAS 2.0 TC-HK domains’ medians ranged from 25 (Do1: Cognition) to 50 (Do2: Mobility, Do5(1): Life Activities – Household), with interquartile ranges (IQRs) varying between 30–60, indicating moderate to high disability levels. SF-36 TC-HK scores showed notable variability, ranging from 25 to 66.67 for role limitations due to physical health (IQR: 75) and role limitations due to emotional problems (IQR: 100), implying the severe effect from the physical aspect and the mild effect from the emotional aspect on role activities. WHOQOL-BREF TC-HK medians were 50 (Physical, Psychological) and 58.33–59.38 (Social, Environment), with narrow IQRs (16.67–23.21), suggesting less variability in quality-of-life perceptions.

**Table 2:** Summary scores and reliability of the WHODAS 2.0 TC-HK, Summary scores of SF-36 TC-HK, and WHOQOL-BREF TC-HK.

Domain	N	Median	IQR	Cronbach's Alpha	Test-retest ICC[a]
**WHODAS 2.0 TC-HK (the higher the scores, the more disability)**					
Do1: Cognition	177	25	30	0.883	0.591
Do2: Mobility	177	50	59.35	0.886	0.872
Do3: Self-care	177	30	40	0.873	0.807
Do4: Getting along	177	33.3	33.3	0.883	0.669
Do5(1): Life activities: household	177	50	60	0.857	0.765[b]
Do5(2): Life activities: work/study	48	28.6	35.7	0.875	0.798
Do6: Participation	177	37.5	33.4	0.869	0.815
**SF-36 TC-HK (the lower the scores, the more disability)**					
Physical functioning	177	45	45		
Role limitations due to physical health	177	25	75		
Role limitations due to emotional problems	177	66.67	100		
Energy/fatigue	177	50	25		
Emotional well-being	177	64	26		
Social functioning	177	50	37.5		
Pain	177	57.5	38.75		
General health	177	45	30		
Health change	177	50	50		
**WHOQOL-BREF TC-HK (the lower the scores, the more disability)**					
Physical	177	50	23.21		
Psychological	177	50	25		
Social Relationships	177	58.33	16.67		
Environment	177	59.38	18.75		

[a]For test-retest, there were 36 patients analyzed in the study

[b]For Life activities: work/study, there were 12 patients analyzed for ICC

All WHODAS 2.0 TC-HK domains demonstrated high internal consistency, with Cronbach’s alpha scores ranging from 0.86 to 0.89, indicating strong reliability from the cut-off of satisfactory score as above 0.8. The highest consistency was observed in the mobility (α = 0.89) and cognition domain (α = 0.88). The lowest, though still excellent, was for life activities: household domain (α = 0.86).

WHODAS2.0 TC-HK has shown moderate to good test-retest reliability (ICC = 0.59–0.87). The highest reliability was found in mobility (ICC = 0.87) and the participation domain (ICC = 0.82). The lowest was the cognition domain (ICC = 0.59), suggesting some response variability over time. For life activities: work/study domain, which was analysed in 12 patients, ICC was 0.80, indicating good reliability despite the smaller sample.

From Table 3, WHODAS2.0 TC-HK domains and SF-36 TC-HK sub scores were negatively and moderately correlated with statistical significance (rs = −0.25 to −0.81, p < 0.05). For the correlations between WHODAS2.0 TC-HK domains and WHOQOL-BREF sub scores, significantly negative and moderate correlations were found as rs = −0.29 to −0.62 in majority of items (Table 4).

**Table 3:** Spearman's correlation coefficients between WHODAS2.0 TC-HK and SF-36 TC-HK.

WHODAS2.0 TC-HK domain/SF-36 TC-HK sub-score	Cognition	Mobility	Self-care	Getting along	Life activities (domestic responsibilities)	Life activities (work & school)	Participation
Physical functioning	−0.003	−0.805^**^	−0.599^**^	−0.188^*^	−0.522^**^	−0.590^**^	−0.370^**^
Role limitations due to physical health	−0.111	−0.405^**^	−0.262^**^	−0.115	−0.345^**^	−0.310^*^	−0.400^**^
Role limitations due to emotional problems	−0.183^*^	−0.110	−0.042	0.004	−0.176^*^	−0.181	−0.346^**^
Energy/fatigue	−0.299^**^	−0.295^**^	−0.191^*^	−0.203^**^	−0.322^**^	−0.173	−0.480^**^
Emotional well being	−0.363^**^	−0.069	−0.080	−0.147	−0.140	−0.140	−0.382^**^
Social functioning	−0.279^**^	−0.294^**^	−0.199^**^	−0.186^*^	−0.239^**^	−0.241	−0.530^**^
Pain	−0.131	−0.268^**^	−0.095	0.034	−0.134	−0.233	−0.250^**^
General health	−0.231^**^	−0.283^**^	−0.265^**^	−0.204^**^	−0.166^*^	−0.287^*^	−0.390^**^
Health change	−0.157^*^	−0.199^**^	−0.147	−0.022	−0.149^*^	−0.411^**^	−0.20^**^

**. Correlation is significant at the 0.01 level (2-tailed).

*. Correlation is significant at the 0.05 level (2-tailed).

**Table 4:** Spearman's correlation coefficients between WHODAS2.0 TC-HK and WHOQOL-BREF TC-HK.

WHODAS2.0 TC-HK domain/ WHOQOL-BREF TC-HK	Cognition	Mobility	Self-care	Getting along	Life activities (domestic responsibilities)	Life activities (work & school)	Participation
Physical health	−0.349^**^	−0.549^**^	−0.491^**^	−0.386^**^	−0.586^**^	−0.469^**^	−0.618^**^
Psychological health	−0.3099^**^	−0.139	−0.168^*^	−0.297^**^	−0.329^**^	−0.318^*^	−0.432^**^
Social relationships	−0.313^**^	−0.072	−0.154^*^	−0.389^**^	−0.291^**^	−0.263	−0.371^**^
Environment	−0.316^**^	−0.156^*^	−0.184^*^	−0.301^**^	−0.225^**^	−0.176	−0.414^**^

**. Correlation is significant at the 0.01 level (2-tailed).

*. Correlation is significant at the 0.05 level (2-tailed).

**Fig 1:**
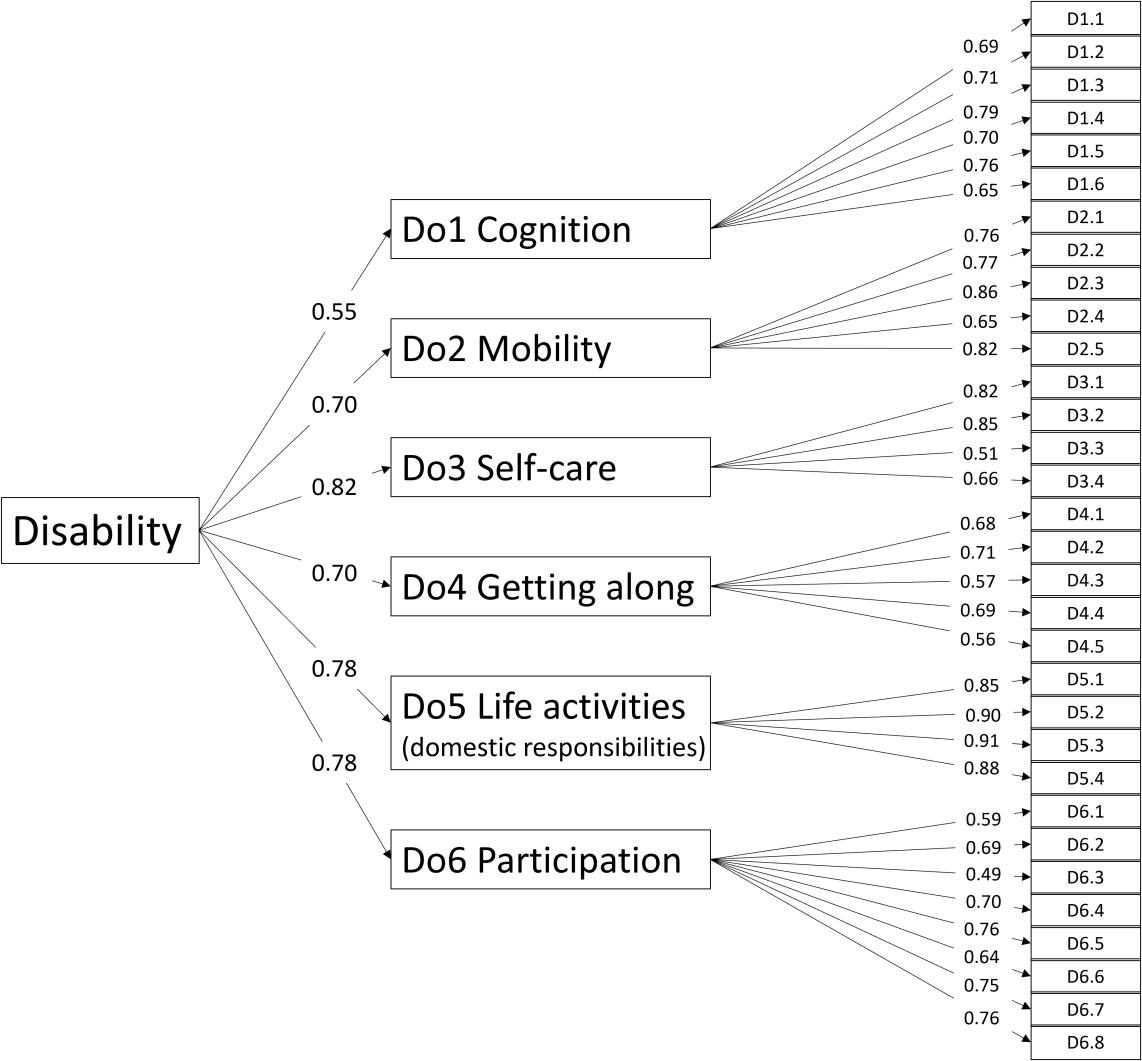
Confirmatory factor analysis (CFA) model of the WHODAS 2.0 TC‑HK (32‑item version).

WHODAS2.0 TC-HK 32-item version, excluding items from Do5(2): Life activities: work/study, was tested in CFA for construct validity. A second-order 6-factor model with the standardized parameter estimates and fit indices was summarized (Fig 1).

The diagram illustrates the second‑order latent construct of disability and six first‑order domains (Cognition, Mobility, Self‑care, Getting along, Life activities – household, and Participation). Standardized factor loadings are shown for each item.

## Discussion

Based on our best knowledge, the present study was the first to develop and validate the Hong Kong Chinese version of WHODAS 2.0. The properties measured in WHODAS 2.0 TC-HK were tested to be a valid and reliable tool in assessing disability for persons with disabilities in the Hong Kong community settings.

Satisfactory internal consistency was found as Cronbach’s alpha was all above 0.8. This was consistent with other studies with similar subjects [8,11,14]. Five domains showed good test-retest reliability (0.77–0.87). The lowest ICC was found in Do 1 Cognition (0.59) and Do 4 Getting along (0.67). In other studies, in Europe, Taiwan, and Norway, the ICC ranged from 0.20–0.74, 0.83–0.89, and 0.63–0.87, respectively [8,9,32]. As ICC was influenced by variability, it was found that Taiwan’s study was using face-to-face interviews in data collection, while the current study, Europe’s study, and Norway’s study were using self-administered questionnaires in data collection [8,9,32]. A similar study in Singapore showed that the ICC in the self-administered version would have a lower value [33]. Moreover, one of the drawbacks in the current study was that the sample size for reproducibility could not reach the recommended minimum(n = 50) [34]. However, regarding the heterogeneity of the current study population, reaching a cut-off of 0.7 for all domains in Cronbach’s alpha and five out of seven domains in ICC indicated acceptable reproducibility for group comparisons in WHODAS2.0 TC-HK [8,27].

This study showed that WHODAS2.0 TC-HK had moderate to good convergent validity with SF36-TC-HK. The majority of domains of the 36-item WHODAS2.0 TC-HK were negatively correlated with domains of the SF-36 TC-HK. The higher the scores (the higher disability) at WHODAS2.0 TC-HK indicated the lower the scores (the lower quality of life) at SF36 TC-HK. The sub-scores of SF36-TC HK all have moderate correlation (rs = −0.20 to −0.53) with participation of WHODAS2.0 TC-HK. It showed the same with other similar studies [8–10]. This was coherent with the concept of ICF as participation being essential in accessing disability [1,2,4].

Furthermore, WHODAS2.0 TC-HK had moderate to good concurrent validity with WHOQOL-BREF TC-HK. WHODAS2.0 TC-HK was negatively and moderately correlated with WHOQOL-BREF TC-HK, as 19 out of 28 correlations rs = −0.3 to −0.57 and p < 0.05. Compared with other studies, a similar result was obtained in Taiwan [32]. It demonstrated that WHODAS2.0 TC-HK was valid and valuable practically. However, it could not express all the meanings in WHOQOL-BREF TC-HK. Indeed, the result was quite consistent with the original study during the development of WHODAS 2.0 in 2010 [6].

For the CFA model, the WHODAS2.0 TC‑HK 32‑item version was analyzed because only 48 participants (27.1%) completed the Do5(2): Life activities: work/study domain. Similar findings regarding limited completion of the work/study items were reported in previous studies conducted in Indonesia and other rehabilitation populations [11]. The CFA results demonstrated suboptimal model fit, indicating that the original six‑factor structure was not fully supported in the present sample. Therefore, the structural validity findings should be interpreted cautiously and regarded as preliminary. Nevertheless, most item factor loadings exceeded 0.54, indicating fair to good relationships between the items and their hypothesized domains. Similar challenges regarding CFA model fit have also been reported in previous cross‑cultural validation studies of WHODAS 2.0 [8,11,32]. The mixed evidence regarding structural validity across populations may reflect cultural, linguistic, and contextual differences influencing disability perception and reporting [35–37].

Recent evidence synthesized from global validation studies similarly suggested that WHODAS 2.0 generally demonstrates satisfactory reliability and convergent validity across diverse populations, while evidence regarding structural validity remains inconsistent across cultural settings and clinical groups [38]. The present findings are therefore broadly consistent with the current international psychometric literature.

There were some limitations in the present study. Sampling size was relatively small when comparing similar studies [8,13,39]. Based on the guideline proposed in 2016, sample sizes less than 100 cases should not be recommended for any type of Structural Equation Modeling [40]. While the sample-to-item ratio should not be less than 5:1 [41]. Therefore, in this study, N = 177 should be considered as the acceptable level. More importantly, it was suggested that a large sample size with blind selection (N > 300) was not more meaningful than a small sample size with careful selection (N > 150) [42]. Secondly, participants recruited were from two geographical regions of Hong Kong and four units held by one NGO. The generalizability of our findings to others living in different regions and receiving services from different NGOs may need further studies. Another limitation was that the assessment tools were in Hong Kong Traditional Chinese. Therefore, for those who were not fluent in Cantonese, the validity and reliability may not be applicable. As 90.6% of the population in Hong Kong were using Cantonese [43], the current findings were useful and applicable to the service users in Hong Kong.

## Conclusions

WHODAS 2.0 TC‑HK demonstrated good internal consistency, acceptable test–retest reliability, and moderate convergent and concurrent validity among adults receiving community rehabilitation services in Hong Kong. While the findings support its potential clinical and service‑level utility, evidence regarding structural validity remains preliminary, particularly because confirmatory factor analysis was conducted using the 32‑item version rather than the full 36‑item instrument. Therefore, WHODAS 2.0 TC‑HK may be considered a useful standardized disability assessment tool for community rehabilitation settings, with appropriate caution regarding interpretation of its factorial structure. Future studies using larger and more diverse samples are recommended to further evaluate the structural validity and cross‑population applicability of the full 36‑item WHODAS 2.0 TC‑HK.

## Supporting information

S1 Table Participants’ demographics and types of disabilities for test-retest. The de-identified dataset generated and analyzed during the current study is provided as Supporting Information in an English-language version in accordance with PLOS ONE data-sharing requirements. Variable definitions and coding descriptions are provided in the accompanying codebook.
(PDF)
