# Supplementary material for: Development and validation of World Health Organization Assessment Schedule 2.0 for persons with disabilities having community rehabilitation services in Hong Kong
Source: PLoS One. 2026 Aug 6;21(8):e0326409. doi: 10.1371/journal.pone.0326409 (PMC13446659; doi:10.1371/journal.pone.0326409)
Supplement: Supplementary file 1 [file pone.0326409.s001.pdf]

36-item version, self-administered

This questionnaire asks about difficulties due to health conditions. Health conditions include diseases or illnesses, other health problems that may be short or long lasting, injuries, mental or emotional problems, and problems with alcohol or drugs.

Think back over the past 30 days and answer these questions, thinking about how much difficulty you had doing the following activities. For each question, please circle only one response.

In the past 30 days, how much difficulty did you have in:						
**Understanding and communicating**						
D1.1	Concentrating on doing something for ten minutes?	None	Mild	Moderate	Severe	Extreme or cannot do
D1.2	Remembering to do important things?	None	Mild	Moderate	Severe	Extreme or cannot do
D1.3	Analysing and finding solutions to problems in day-to-day life?	None	Mild	Moderate	Severe	Extreme or cannot do
D1.4	Learning a new task, for example, learning how to get to a new place?	None	Mild	Moderate	Severe	Extreme or cannot do
D1.5	Generally understanding what people say?	None	Mild	Moderate	Severe	Extreme or cannot do
D1.6	Starting and maintaining a conversation?	None	Mild	Moderate	Severe	Extreme or cannot do
**Getting around**						
D2.1	Standing for long periods such as 30 minutes?	None	Mild	Moderate	Severe	Extreme or cannot do
D2.2	Standing up from sitting down?	None	Mild	Moderate	Severe	Extreme or cannot do
D2.3	Moving around inside your home?	None	Mild	Moderate	Severe	Extreme or cannot do
D2.4	Getting out of your home?	None	Mild	Moderate	Severe	Extreme or cannot do
D2.5	Walking a long distance such as a kilometre [or equivalent]?	None	Mild	Moderate	Severe	Extreme or cannot do

36
Self

In the past 30 days, how much difficulty did you have in:						
**Self-care**						
D3.1	Washing your whole body?	None	Mild	Moderate	Severe	Extreme or cannot do
D3.2	Getting dressed?	None	Mild	Moderate	Severe	Extreme or cannot do
D3.3	Eating?	None	Mild	Moderate	Severe	Extreme or cannot do
D3.4	Staying by yourself for a few days?	None	Mild	Moderate	Severe	Extreme or cannot do
**Getting along with people**						
D4.1	Dealing with people you do not know?	None	Mild	Moderate	Severe	Extreme or cannot do
D4.2	Maintaining a friendship?	None	Mild	Moderate	Severe	Extreme or cannot do
D4.3	Getting along with people who are close to you?	None	Mild	Moderate	Severe	Extreme or cannot do
D4.4	Making new friends?	None	Mild	Moderate	Severe	Extreme or cannot do
D4.5	Sexual activities?	None	Mild	Moderate	Severe	Extreme or cannot do
**Life activities**						
D5.1	Taking care of your household responsibilities?	None	Mild	Moderate	Severe	Extreme or cannot do
D5.2	Doing most important household tasks well?	None	Mild	Moderate	Severe	Extreme or cannot do
D5.3	Getting all the household work done that you needed to do?	None	Mild	Moderate	Severe	Extreme or cannot do
D5.4	Getting your household work done as quickly as needed?	None	Mild	Moderate	Severe	Extreme or cannot do

If you work (paid, non-paid, self-employed) or go to school, complete questions D5.5–D5.8, below. Otherwise, skip to D6.1.

Because of your health condition, in the past 30 days, how much difficulty did you have in:						
D5.5	Your day-to-day work/school?	None	Mild	Moderate	Severe	Extreme or cannot do
D5.6	Doing your most important work/school tasks well?	None	Mild	Moderate	Severe	Extreme or cannot do
D5.7	Getting all the work done that you need to do?	None	Mild	Moderate	Severe	Extreme or cannot do
D5.8	Getting your work done as quickly as needed?	None	Mild	Moderate	Severe	Extreme or cannot do

**Participation in society**						
In the past 30 days:						
D6.1	How much of a problem did you have in joining in community activities (for example, festivities, religious or other activities) in the same way as anyone else can?	None	Mild	Moderate	Severe	Extreme or cannot do
D6.2	How much of a problem did you have because of barriers or hindrances in the world around you?	None	Mild	Moderate	Severe	Extreme or cannot do
D6.3	How much of a problem did you have living with dignity because of the attitudes and actions of others?	None	Mild	Moderate	Severe	Extreme or cannot do
D6.4	How much time did you spend on your health condition, or its consequences?	None	Mild	Moderate	Severe	Extreme or cannot do
D6.5	How much have you been emotionally affected by your health condition?	None	Mild	Moderate	Severe	Extreme or cannot do
D6.6	How much has your health been a drain on the financial resources of you or your family?	None	Mild	Moderate	Severe	Extreme or cannot do
D6.7	How much of a problem did your family have because of your health problems?	None	Mild	Moderate	Severe	Extreme or cannot do
D6.8	How much of a problem did you have in doing things by yourself for relaxation or pleasure?	None	Mild	Moderate	Severe	Extreme or cannot do
